# 9.3 PSYCHOSIS BIOTYPES VERSUS CLINICAL SYNDROMES THROUGH THE PRISM OF INTRINSIC NEURAL ACTIVITY

**DOI:** 10.1093/schbul/sby014.031

**Published:** 2018-04-01

**Authors:** Brett Clementz, Godfrey Pearlson, Carol Tamminga, John Sweeney, Matcheri Keshavan

**Affiliations:** 1 University of Georgia; 2 Olin Neuropsychiatry Research Center; 3 University of Texas Southwestern Medical Center; 4 Beth Israel Deaconess Medical Center & Harvard Medical School

## Abstract

**Background:**

Deviation in level of intrinsic neural activity (ongoing brain signals recorded with EEG/MEG) is observed in psychosis. Neurophysiological models have proposed this physiological indicator as a genetically mediated core deviation in psychosis. Translational models of intrinsic activity deviations promise to identifying multiple distinct physiological mechanisms for psychosis manifestation. Intrinsic activity deviations may masquerade as higher levels of neural response in sensory cortices, but ultimately may lead to poor signal-to-noise ratios, particularly when psychosis cases are required to identify stimulus salience.

Why do we not hear more about intrinsic activity as a core biomarker for any psychosis variation? An explanation is provided by the current project. The Bipolar-Schizophrenia Network for Intermediate Phenotypes (B-SNIP) published a means for categorizing psychoses by neurobiological homology via use of multiple biomarkers (psychosis Biotypes) rather than by clinical features. B-SNIP demonstrated the superiority of Biotypes versus DSM diagnoses for capturing neurobiological similarity through multiple external validating measures (social functioning, measures of brain volume from structural magnetic resonance images, clinical diagnoses and biomarker features among first-degree relatives). Independent analyses since the initial publication have provided additional support for the usefulness of psychosis Biotypes.

**Methods:**

For this project, we analyzed ongoing neural activity from 64 EEG sensors during 150 intervals of 10 sec duration from over 1450 B-SNIP subjects. These data (never before published) were from the inter-trial interval (ITI) of an auditory paired-stimuli task used in Biotypes construction (these ITI data themselves were not used). Although the subjects were engaged in a task (counting the number of stimulus pairs), the data used here were not part of the task itself. Data were evaluated for single trial power (estimate of neural response strength on individual trials) as a function of frequency of neural oscillations (from 2–50 Hz) over the whole head. Data were then averaged over single trials to yield an estimate of the overall strength of nonspecific (unrelated to sensory processing) neural activity.

**Results:**

When evaluated by DSM diagnoses (schizophrenia, schizoaffective disorder, bipolar disorder), the 95% confidence intervals for all groups overlapped the healthy group means across all frequencies. When considered by psychosis Biotypes, differences were obvious and statistically significant. In comparison to healthy persons, Biotype-2 probands (the most neurophysiologically activated subgroup in previous analyses) were notably high on nonspecific neural activity, and Biotype-1 probands (the most cognitively and neurophysiologically compromised subgroup in previous analysis) were notably low. Group separations on this metric were better than those obtained with original intrinsic EEG measure used in psychosis Biotypes construction, indicating this more pure intrinsic activity measure is capturing a meaningful component of Biotype neurophysiology. This was true across a range of oscillatory frequencies for the probands. The first-degree relatives of the Biotype probands showed similar patterns, although higher frequency oscillations (above 20 Hz) better differentiated relatives from healthy persons.

**Discussion:**

Intrinsic activity deviation is a promising biomarker for translational research programs aimed at differential treatment development, but using DSM psychosis diagnoses would obscure its importance for understanding psychosis.

